# The complete chloroplast genome of *Chamaecyparis obtusa* (Cupressaceae)

**DOI:** 10.1080/23802359.2020.1811793

**Published:** 2020-09-01

**Authors:** Sang-Chul Kim, Jei-Wan Lee

**Affiliations:** Division of Forest Bioinformation, National Institute of Forest Science, Suwon-Si, Republic of Korea

**Keywords:** *Chamaecyparis obtusa*, Cupressaceae, complete chloroplast genome, phylogenetic analysis

## Abstract

The complete plastid genome of *Chamaecyparis obtusa* was sequenced and analyzed in this study. It was found to be 126,821 bp long. The guanine-cytosine content of the whole genome was 35.1%, and there were 83 unique protein-coding genes, 33 tRNAs, and 4 rRNAs. Furthermore, an overlap of 120 bp was found (including *trnQ*-UUG). The order and organization of these genes were consistent with those of other complete plastid genomes from the Cupressaceae. A phylogenetic tree was constructed according to the 83 protein-coding genes found, which demonstrated that *C. obusa* had sister relationships within the genus *Chamaecyparis*.

*Chamaecyparis obtusa* (Siebold & Zucc.) Endl. is an evergreen coniferous tree that is native to Japan, and it can grow up to 30–40 m in height and 50–150 cm in width. The resources of this economically important species were introduced in South Korea from Japan in the 1920s, and they were subsequently planted in the southern provinces of South Korea (Park et al. 2000). Recently, this species has been shown to have antibacterial and anti-inflammatory effects and has been used as a skin-protective agent (Hyun et al. 2016). There are seven species of the genus *Chamaecyparis* worldwide, among which *Chamaecyparis formosensis* (LC177668)*, C. hodginsii* (KX832623), and *C. lawsoniana* (KX832622) have been found to have three types of chloroplast DNA as reported by the National Center for Biotechnology Information. In this study, we have reported, for the first time, the complete sequence of the chloroplast genome of *C. obtusa* and propose a molecular phylogenetic relationship between the chloroplasts of *C. obtusa* and that of other Cupressaceae members.

Fresh leaves were collected from Gangjin-eup, Gangjin-gun, Jeollanam-do, Republic of Korea (E 127°17′ 23.6″, N 34°8′38.9″) and the specimens were deposited at the Warm Temperate and Subtropical Forest Research Center (Accession number: WTFRC-10032794). Genomic DNA was isolated from fresh leaves using a Plasmid SV mini kit (GeneAll, Seoul, Korea). The extracted DNA were stored in the Plant DNA Bank at the National Institute of Forest Science (No. 0335194701; Suwon, Korea). Whole-genome sequencing was conducted using the Ion Torrent sequencing platform (Life Technologies, Carlsbad, CA). Filtered sequences were assembled using *C. lawsoniana* as the reference sequence (GenBank accession number KX832622) in Geneious R10 (Biomatters Ltd, Auckland, New Zealand; Kearse et al. 2012). Annotations were created using DOGMA (http://dogma.ccbb.utexas.edu/) and BLAST searches. All of the tRNA sequences were confirmed using the web-based tool tRNAScan-SE (Schattner et al. 2005) with its default settings to corroborate the tRNA boundaries identified using Geneious. Maximum likelihood (ML) tree searches and ML bootstrap searches were performed using RAxML BlackBox web-server (Stamatakis et al. 2008) with 83 protein-coding genes from 13 Cupressaceae plants. The RAxML analyses were run with a rapid bootstrap analysis using a random starting tree and 100 ML bootstrap replicates.

The plastid of *C. obtusa* was found to have double-stranded, circular DNA of 126,821 bp (GenBank accession number MT258872). The genome contained 120 genes, including 83 protein-coding genes, 33 tRNA genes, and 4 rRNA genes. In addition, an overlap of 120 bp, including *trnQ*-UUG, was found, and the overall GC content was 35.1%. The chloroplast of this species was very similar to that of other species of the Cupressaceae. The *Chamaecyparis* genus was found to be monophyletic (100% bootstrap values, BS). Our results for the complete plastid genome sequence of *C. obtusa* may contribute to a better understanding of the evolution of *Chamaecyparis* (Figure 1) and provide a useful resource for the development of species identification markers.

**Figure 1.**
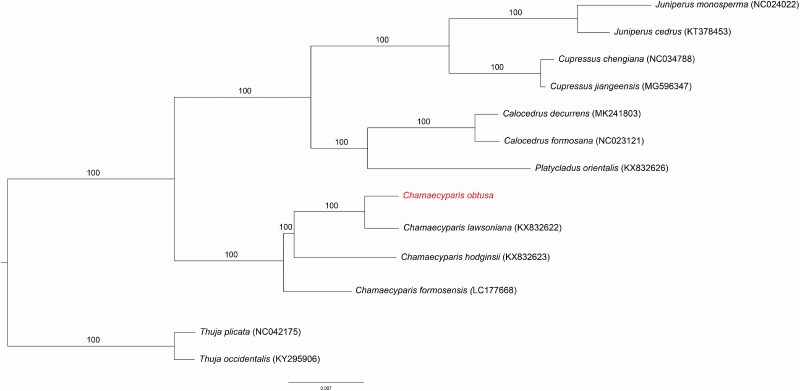
Phylogenetic trees of 14 Cupressaceae species. The tree was generated by maximum likelihood. GenBank accession numbers are shown in the figure.

## Data Availability

The data that support the findings of this study are openly available in The GenBank at https://www.ncbi.nlm.nih.gov/genbank/, reference number “MT258872”.
